# Nanostructured Guidance for Peripheral Nerve Injuries: A Review with a Perspective in the Oral and Maxillofacial Area

**DOI:** 10.3390/ijms15023088

**Published:** 2014-02-20

**Authors:** Stefano Sivolella, Giulia Brunello, Nadia Ferrarese, Alessandro Della Puppa, Domenico D’Avella, Eriberto Bressan, Barbara Zavan

**Affiliations:** 1 Department of Neurosciences, Institute of Clinical Dentistry,University of Padova, Via Venezia, 90, 35129 Padova, Italy; E-Mails: stefano.sivolella@libero.it (S.S.); giulia-bru@libero.it (G.B.); medea_f@hotmail.com (N.F.); eriberto.bressan@unipd.it (E.B.); 2 Department of Neurosciences, University of Padua, via Giustiniani, 5, 35128 Padua, Italy; E-Mails: alessandro.dellapuppa@sanita.padova.it (A.D.P.); domenico.davella@unipd.it (D.D.); 3 Department of Biomedical Sciences, University of Padova, Via G. Colombo 3, 35100 Padova, Italy

**Keywords:** nanofiber, scaffold, nerve regeneration, electrospinning, self-assembly, SAPNS

## Abstract

Injury to peripheral nerves can occur as a result of various surgical procedures, including oral and maxillofacial surgery. In the case of nerve transaction, the gold standard treatment is the end-to-end reconnection of the two nerve stumps. When it cannot be performed, the actual strategies consist of the positioning of a nerve graft between the two stumps. Guided nerve regeneration using nano-structured scaffolds is a promising strategy to promote axon regeneration. Biodegradable electrospun conduits composed of aligned nanofibers is a new class of devices used to improve neurite extension and axon outgrowth. Self assembled peptide nanofibrous scaffolds (SAPNSs) demonstrated promising results in animal models for central nervous system injuries, and, more recently, for peripheral nerve injury. Aims of this work are (1) to review electrospun and self-assembled nanofibrous scaffolds use *in vitro* and *in vivo* for peripheral nerve regeneration; and (2) its application in peripheral nerve injuries treatment. The review focused on nanofibrous scaffolds with a diameter of less than approximately 250 nm. The conjugation in a nano scale of a natural bioactive factor with a resorbable synthetic or natural material may represent the best compromise providing both biological and mechanical cues for guided nerve regeneration. Injured peripheral nerves, such as trigeminal and facial, may benefit from these treatments.

## 1. Introduction

The expectation of humans to live long lives demands continuous development of new therapeutic strategies that can promote the regeneration of tissues damaged by trauma, disease, or congenital defects. The field of regenerative medicine aims to meet these demands, focusing on restoring lost, damaged, aged, structures to return function to tissues. There are many targets where new therapies could greatly improve both the span and quality of life. In some conditions, normal physiologic regeneration is limited or non-existent [1]. The ability to regenerate the peripheral nerve damage could generate sensory dysfunctions, which, if persistent or painful, can be distressing to both the patient and the clinician.

Between peripheral nerve damages, those regarding the oral and maxillofacial area may results from acute trauma, progressive nerve compression or degenerative diseases. The nerves that can be damaged in this specific area are facial, inferior alveolar, mental, lingual, incisal, nasopalatine, greater palatine, and infraorbital nerves [2–10]. The most commonly involved is the mandibular nerve. Injury to the branches of the mandibular division of the trigeminal nerve (inferior alveolar nerve, lingual nerve, and mental nerve) is a frequent complication in implant surgery or bone grafting procedures [11]. Injury of the inferior alveolar and lingual nerves can be related to third molar surgery as well [12]. A trigeminal nerve injury can occur as a consequence of several factors, including expanding compressing lesions, such as benign or malignant tumors and cystic lesions, local infections, and iatrogenic lesions during anesthetic injection, tooth extraction, overfilling of the canals in endodontic therapy, flap elevation, apical surgery, nerve transposition, orthognathic and pre-prosthetic surgery.

Surgical procedures for repairing injured peripherical nerves are indicated in case of axonotmesis and neurotmesis, according to Seddon classification [13]. They consist of direct end-to-end reconnection, based on the direct coaptation of the transacted nerve stumps (microsurgical neurorraphy), or the use of nerve grafts in larger nerve defects, such as the sural nerve and greater auricular nerve [14,15] in case of trigeminal branches damage. Autografting presents some critical drawbacks, including limited availability of donor graft and mismatch in size. Additional surgery trauma at donor sites and associated functional loss of a donor nerve are undesirable adverse consequences [16]. Allografts and xenografts are also taken into consideration as alternatives to autologous nerve grafts. However, the patient is at a risk of immunological reactions and disease transmission [17,18]. Another strategy may consist of the utilization of processed human decellularized allograft product [19].

Guided nerve regeneration is a promising strategy to promote axon regeneration. It consists of placement of a material to connect the lesion gap to guide axonal sprouting and regeneration across a nerve gap from proximal to distal portions of a nerve. In this view, a variety of studies have investigated nanostructured devices as guidance for peripheral nerve injuries [14,20].

Nanotechnology reaches and develops manipulation under control of the nanoscale structures, in the length scale of approximately 1–100 nanometer range, and their integration into larger material components, systems and architectures. In some particular cases, the critical length scale for novel properties and phenomena may be under 1 nm or be larger than 100 nm [21]. In the field of tissue engineering, the term “nanofiber” is typically used to describe fibers with diameters ranging from 1 to 1000 nm [22].

Nanomaterials closely mimic the extracellular matrix (ECM), allowing them to be used as biomimetic scaffolds. Moreover, the high surface area-to-volume ratio is ideal for cell attachment and drug loading. Additionally, nanofibers have been shown to display unique mechanical properties and higher rates of selective protein adsorption [23].

The production of polymeric nanofibers for regenerative medicine can be obtained trough different methods. Two widely-studied fiber-fabrication methods commonly applied in neural regeneration are electrospinning and self-assembly. Macroscale tubes based on electrospun and self-assembled nanofibers seem to be promising candidates for application in peripheral nerve regeneration.

Aim of this review is to evaluate the applications of nanostructured biomaterials for peripheral nerve regeneration, with a perspective to the oral and maxillofacial surgery fields.

## 2. Electrospun Nanofibers for Neural Tissue Engineering

### 2.1. Method of Production and Major Characteristics

Electrospinning is a technique capable of generating fibers with diameters down to the nanoscale. A non-woven mat of electrospun nanofibers possesses high porosity and spatial interconnectivity well-suited for nutrient and waste transport and cell communication. A scaffold based on electrospun nanofibers also has a large specific surface area for loading of bioactive molecules to facilitate efficient and selective cellular responses.

Four major components are required for electrospinning a spinneret (e.g., a hypodermic needle with blunt-tip), a syringe pump for ejecting the polymer solution at a controlled rate, a direct current (DC) power supply up to 30 kV, and a grounded collector. When the polymer solution emerges from a spinneret, it initially forms a droplet due to the confinement of surface tension. If a high voltage is applied to the spinneret, charges of the same sign will be built on the surface of the droplet. Once the repulsion among the charges is sufficiently strong to overcome the surface tension, a Taylor cone will be formed, followed by a liquid jet directed towards the grounded collector. The jet will experience both solvent evaporation and whipping instability before it reaches the collector. As a result of stretching by electrostatic repulsion and whipping, the liquid jet will be continuously reduced in size until it has been solidified or deposited on the collector. By adjusting experimental parameters such as the concentration of polymer solution, the voltage, and the distance between spinneret and collector, fibers with uniform diameters can be routinely produced [23,24].

The fiber orientation can be directed using different types of collectors, instead of a flat plate, in order to manipulate the distribution of the electric field, such as a rotating mandrel, rapidly oscillating frame, a ring electrode, a metal frame, a rotating drum, and a pair of electrodes separated by an insulating gap [14,25].

With this method, it is possible to control many factors, such as the fiber diameter, alignment, and composition. In addition, electrospinning is applicable to a wide variety of polymers that can be derived from natural sources or synthesized. It does not involve heating or chemical reactions during tube synthesis, allowing materials that are not stable to heat or chemical reactions to be processed into microfibrous or nanofibrous form. Disadvantages include the use of organic solvents and the limited control of pore structures [20,23,26].

Nanofiber electrospun scaffolds might be interesting devices in neural tissue engineering not only for their capability in mimicking the fibrous components of the neural ECM, but also for the possibility of delivering neurotrophic factors to the site of injury. In fact, this technique allows the incorporation of bioactive factors into the scaffolds to guide and enhance neurite extension and axon regrowth, incorporating biochemical and topographical cues into a single scaffold [27].

The materials used in nanostructured electrospun scaffolds may be categorized into three main groups:

#### 2.1.1. Electrospun Synthetic Materials

Electrospun synthetic materials are suitable for constructing nanostructured scaffold for neural tissue engineering, due to their mechanical properties and the ease in tailoring the degradation rate [27]. Main materials investigated are: polyesters such as poly(glycolic acid) (PGA), poly(l-lactic acid) (PLLA) [28], poly(caprolactone) (PCL) [29], poly(3-hydroxybutyrate) (PHB) [30], poly(3-hydroxybutyrate-co-3-hydroxyvalerate) (PHBV) [30] and their copolymers, polydioxanone (PDS) [31], poly acrylonitrile-co-methylacrylate (PAN-MA) [32]. Polyesters are the most extensively investigated electrospun synthetic polymers to induce neural growth; they are characterized by biodegradability and hydrophobic properties. Even though they show good mechanical properties and minimal toxicity to the host, their hydrophobic component leads to unsatisfactory cell interactivity [20,27] (Table 1).

#### 2.1.2. Electrospun Natural Materials

Electrospun natural materials are characterized by similar mechanical and physical properties to the damaged tissue, but immunogenicity and high cost limit their use. One of the more frequently used is collagen, which is one of the most represented components of the ECM and connective tissue. Also silk fibroin, gelatin, laminin and chitosan could be used for the production of nanofibrous scaffolds [33–37].

#### 2.1.3. Electrospun Biosynthetic Materials

Electrospun biosynthetic materials nanofibrous constructs can be loaded with bioactive factors, combining synthetic and natural materials, with improved mechanical properties over natural polymers and enhanced biocompatibility over synthetic ones. Proteins, small proteins and nucleic acid can blended into the polymer solution during electrospinning. Other methods employed to add bioactive factors are coaxial electrospinning, immobilization and adsorption techniques. The elecrospun nanofibers can be funzionalized by the adjunction of ECM proteins and peptides (e.g., collagen, laminin, fibronectin, gelatin), polysaccharides (e.g., chitosan), neuroactive peptides (e.g., human tenascin), or growth factors (e.g., nerve growth factor, NGF, basic fibroblast growth factor, bFGF) [14,23,27].

Electrospun nanomaterials have been extensively studied *in vitro* and *in vivo*. Topographical (fiber diameter and orientation) and biochemical (natural bioactive factor loading) cues can be added to such materials to improve their guidance effects.

In the next paragraphs, the mechanisms of action of electrospun nanofibers for neural tissue engineering and the main outcomes of in vivo and in vitro studies will be described in detail.

Main features of nanostructured electrospun materials used *in vitro* and *in vivo* studies are summarized in Table 2 and Table 3, respectively.

### 2.2. *In Vitro* Studies

#### 2.2.1. Nanostructured Electrospun Scaffold Architecture: Topographical Cues

In the case of transected peripheral nerves, the regenerative process might be promoted surgically bridging the gap between the proximal and distal stumps of the injured nerve with a conduit acting as an artificial device supporting axonal growth and guidance. These conduits should direct axonal sprouting, prevent the growth of fibrous tissue into the defects, and promote the diffusion of neurotrophic factors [23].

Nanostructured electrospun scaffolds provide an alternative approach for neural regeneration, mimicking extracellular matrix (ECM) topography and due to their ability to promote neurite outgrowth. Several properties of nanofiber scaffolds influence cell proliferation and differentiation *in vitro*. The arrangement of electrospun nanofibers influences the growth patterns of cells of both neuronal and glial origin. Compared to randomly oriented nanofibers, electrospun aligned nanofibers might provide much better guidance cues in nerve tissue engineering. The fiber diameter played a vital role in nerve regeneration, with better performances detected with smaller diameter fibers. Electrical cues resulted to be critical topic as well.

In order to determine the ideal nano-patterned substrate with the best mechanical and biological properties for nerve tissue engineering, many random and aligned nanofibers, the two main categories in which electrospun scaffolds can be divided, have been extensively tested *in vitro*.

Yang *et al*. [38] produced electrospun nanofibrous poly(l-lactic acid) (PLLA) scaffold as biocompatible and degradable substrates for neural tissue engineering. *In vitro* studies were performed using neural stem cells (NSCs) with good results: the nanostructured porous scaffold composed of randomly oriented fibers promoted neural stem cell differentiation and adhesion and supported neurite outgrowth.

The same authors [39] tested random and aligned PLLA nano/micro fibrous scaffolds for nerve regeneration. They found that cell differentiation rate of neural stem cell C17.2 was higher on nanofibers than on microfibers, independent of the fiber alignment, but strictly related to fiber diameters. Moreover, aligned electrospun scaffolds directed neurite extension of NSCs parallel to the direction of PLLA fibers, with no significant changes with respect to the fiber diameters. The authors concluded that nanofibrous aligned PLLA scaffolds could be use as potential stem cell carriers to the injured nerve sites.

PLLA aligned electrospun nanofibers without any surface modification could direct neurite growth from dorsal root ganglia (DRG) [40]. The correlation between fiber alignment (high, intermediate, and random) and neurite alignment was studied performing Fourier image analysis. The authors found that highly aligned fibers produced significantly highly aligned neuritis, as compared to fiber of intermediate or random alignment, and fibers of intermediate alignment were superior to those with random alignment. In addition, they observed that highly aligned fibers increased the rate of neurite growth by 20% and 16% compared to fibers of randomly and intermediate alignment, respectively. The increased speed reported in neurite growth on highly aligned fibers was consistent with that seen in C17 cells by Yang *et al*. [39]. The radial neurite growth from DRG appeared to be directed along the fibers upon or soon after neurite contact, confirming that neurite growth was superior when neurites encounter anisotropic cues. The shape of the ganglia shape was also affected by fiber alignment, with ganglia elongating along the fibers. Some DRG were stained with antibody to s100 (glial marker) to investigate Schwann cell behavior, it resulted that the fibers caused the alignment of Schwann cells and provided a topographical guidance cue for neurites. Finally, they used two well-characterized human neuroblastoma cell lines to test the influence of aligned fibers on individual cells that did not require adhesive coatings, finding orientated cell morphology when seeded on aligned PLLA fibers. Another resorbable material, electrospun scaffold, polydioxanone (PDS) possessing either aligned or randomly oriented fibers was studied [31]. DRG neurons grown on random electrospun PDS matrices showed no directional preference, whereas neurites grown on aligned matrices displayed directionality that mimics that of the underlying fiber orientation. Cells of glial origin were tested; the findings confirmed the potential of aligned matrices to influence growth dynamics of astrocytes. Authors hypothesized that a glial substrate might provide a more stable, supportive interface between the resorbable matrix and the outgrowing neuritis, especially for central nervous system (CNS) repair. Accordingly to this aim, DRG were co-cultured with astrocytes that were first seeded on matrices, DRG cultured on a substrate of astrocytes resulted to grow more robustly and extend longer processes than when grown on a glia-free matrix. For this reason Subramanian and co-workers’ study [41] developed a neural scaffold that possessed both electrical and topographical cues. They fabricated and compared 2D random and 3D axially aligned electrically conducting biodegradable scaffold obtained from poly(lactide-*co*-glycolide) (PLGA)/poly(3-hexylthiophene) (PHT) blending. The pore size of random PLGA-PHT nanofibers and Young’s modulus resulted significantly higher than in the aligned samples. Aligned nanofibers showed significantly lesser degradation rate and higher electrical conductivity than random nanofibers. The scaffolds were characterized for cell adhesive and proliferative properties *in vitro* using Schwann cells. The morphology of adhered cells on both random and aligned PLGA-PHT scaffolds were evaluated using SEM and laser scanning confocal microscope; longitudinally aligned scaffold provided the specific topographical cue to orient the cells along the fiber direction. In addition, the authors supposed that the higher glial cell adhesion and proliferation in aligned samples could be attributed to the 3D structure of the aligned nanofibers, which mimicked the architecture of endoneural process of peripheral nerves *in vivo*. The authors concluded that longitudinally aligned 3D scaffolds could be potential candidates as conduit for neural regeneration, having satisfying flexibility, good electrical property, and slow degradation rate. Their results were consistent with the observations reported in other studies [29,49], which highlighted the influence of nanofiber arrangement on cell proliferation and growth patterns.

Natural polymers have been investigated as well. Nanofiber diameter seems to influence neuronal repair and regeneration. Qu and colleagues [37] *in vitro* investigated the effects of electrospun biodegradable silk fibroin (SF) scaffold-diameter in regulating and directing cell behaviors. To assess the effects of SF scaffolds-diameter on cell growth and development, neurons and astrocytes were collected and cultured separately or in combination on SF scaffolds in different diameters (400, 800, and 1200 nm). βIII-tubulin immunofluorescence revealed that primary dendrite length, the total dendrite length, and the number of dendrite branches of neurons on 400 nm SF scaffolds were much longer than that on larger-diameter scaffolds, indicating the favorable role of smaller diameter scaffolds to the growth and maturation of neurons. Based on GFAP (glial fibrillary acidic protein) immunofluorescence staining observations, astrocytes on SF scaffolds with smaller diameters exhibited increased cell spreading. Moreover, at time-lapse video analysis astrocytes on SF scaffolds showed significant increase in migration efficiency, as compared with controls (poly-l-lysine substrates). The authors highlighted the essential role of astrocytes to support and promote nerve tissue repair; the increase in migration efficiency of astrocytes grown on SF was considered an important device strategy, in order to limit the formation of glial scars and prompt beneficial astrocytic repair following nerve injury.

Nanofiber orientation was found to have a significant role in determining cell growth and orientation, with better results when aligned nanofibers had been applied.

Consistent with the previous study [37], Wang *et al*. [42] found that the scaffold topography, included the nanofiber diameter, was involved in regulating cell growth and differentiation. Human embryonic stem cell-derived neural precursors (hESC-derived NPs) were cultured on electrospun Tussah silk fibroin (TSF)-substrates of different diameter (400 and 800 nm) and orientation (random and aligned) for 7 days in neural differentiation medium, to explore the effect of fiber topography on cell viability, neuronal differentiation, and neurite outgrowth. They found significant improvement of neuronal differentiation and neurite outgrowth in aligned TSF-scaffold samples, compared with random ones. Moreover, on aligned 400 nm fibers cell viability, neuronal differentiation, and neurite outgrowth were greater than those on aligned 800 nm fibers, indicating that the TSF-scaffold composed of oriented nanofibers of smaller diameter was the most promising scaffold to guide nerve regeneration.

The substratum impact, in particular fiber orientation, on proliferation on spinal cord derived neural progenitor cells (NPCs) was investigated by Wang and colleagues [43]. To reach their aim, the employed random and aligned collagen type I nanofibrous scaffolds to mimic native matrix. Collagen as a native component of ECM provided an ideal scaffold, promoting NPCs proliferation. Moreover, NPCs expanded faster on aligned nanofibers than that cultured on randomly oriented nanofibers, indicating the crucial role of fiber alignment in cell expansion. Finally, they examined cell cycle dynamics of NPCs and the intrinsic mechanisms behind the effects of nanofibrous scaffolds on these kind of cells. They demonstrated that β1 integrin interacted with collagen nanofibers and activated ERK1/2, which in turn modulated cyclin D1 and CDK2 expression and thus controlled cell cycle progression.

#### 2.2.2. Bioactive Factor Loaded on Nanostructured Electrospun Scaffolds: Biochemical Cues

Nanofiber constructs are suitable for neural tissue regeneration not only for their structure characteristics mimicking the fibrous components of the neural ECM, but also because of the possibility of loading these scaffolds with bioactive factors that can be delivered in the site of injury [59]. Many copolymers, composed of nanostructured natural or synthetic polymers and a bioactive factor loaded, have been investigated. This strategy aim to enhance biomechanical properties and cell affinity of the macromolecules obtained. Three different methods of incorporating an extracellular protein into synthetic electrospun fibers (covalent binding, physical adsorption and blended electrospinning procedures) were described by Koh [28] to create a biomimetic scaffold for peripheral nerve repair. Laminin resulted successfully added onto poly(l-lactic acid) (PLLA) nanofibers. In particular, PC12 cell viability and neurite outgrowth assays revealed that blended electrospinning of laminin and synthetic polymer was the easiest and most efficient method to functionalize PLLA nanofibers.

Laminin addition to a synthetic polymer was also performed by Christopherson and co-workers [44], who analyzed the influence of both topographical (specifically nanofiber diameter) and biological (laminin addition) features on cell proliferation and differentiation. They cultured rat hippocampus-derived adult NSCs (rNSCs) on laminin-coated electrospun Polyethersulfone (PES) fiber meshes with different fiber diameters to investigate the effects of fiber diameters (283 ± 45, 749 ± 153, 1452 ± 312 nm) in regulating and directing cell behaviors. They found that as the fiber diameter increased, rNSCs showed reduced migration, spreading and proliferation in the presence of FGF-2 and serum free medium. Under differentiation conditions, rNSCs spread and assumed glial cell shape and preferentially differentiated into oligodendrocytes, whereas they elongated on larger diameter fibers and preferentially differentiated into neuronal lineage. They concluded that topographical cues, when applied in combination with targeted biochemical signals, might be an instructive tool to regulate the lineage specification of stem cells. The effects of a bioactive factor inclusion to synthetic electrospun fibers on neural cell behavior were also evaluated by Ahmed *et al*. [45]. They tested *in vitro* a surface-modified polyamide nanofibers by covalent attachment of neuroactive peptides derived from human tenascin-C. These peptides were found to enhance the ability of the nanofibers to facilitate neuronal attachment, neurite generation and extension.

Biosynthetic electrospun nanofibers were also fabricated by Xie *et al*. [46], who investigated neurite outgrowth on PCL nanofiber scaffolds with different orders, structures, and surface properties. They found that dorsal root ganglia (DRG) cultured on random nanofibers presented neurites extended radially without specific direction. The neurites preferentially extended along the long axis of fibers, when cultured on aligned nanofibers. When seeded at the border between aligned and random fibers, the neurites originating from the same DRG could simultaneously expressed aligned and random neurite fields in response to the underlying nanofibers. In addition, when cultured on a double-layered scaffold, neurite outgrowth pattern was found to be determined by fiber orientation in both layers. The biological properties of PCL, could be enhanced by the addition of laminin coating, as demonstrated in this study.

A modified PCL nanofiber scaffold (PCL poly(ɛ-caprolactone)/gelatin scaffold) was studied by Ghasemi-Mobarakeh [29]. Incorporation of gelatin, a natural material derived from collagen hydrolysis, improved the hydrophilicity of PCL/gelatin nanofibrous scaffolds. Electrospun electrospun PCL/gelatin nanofibers with ratio of 50:50 had fastest degradation rate and weakest mechanical properties, which may not be favorable for nerve regeneration. The use of PCL/gelatin with ratio of 70:30 was found to exhibit the most balanced properties for nerve tissue engineering and was selected for *in vitro* experiments. It was found that PCL/gelatin 70:30 enhanced differentiation and proliferation of nerve stem cells (C17.2 cells) compared to PCL nanofibrous scaffolds. Finally, they observed that aligned nanofibers highly supported the nerve cells and improved the axon outgrowth and cell differentiation process.

Prabhakaran and colleagues [47] investigated PCL/chitosan nano-scaled fibrous scaffolds *in vitro* using rat Schawann cells for nerve regeneration. They blended PCL with chitosan using electrospinning process. They found that PCL/chitosan nanofibers presented a narrow distribution of fiber diameter and a higher hydrophilicity in comparison with PCL nanofibers. In addition, mechanical properties of electrospun PCL/chitosan nanofibers were better than those of the chitosan nanofibers. Moreover, the MTS assay revealed significantly higher proliferation rate of rat Schwann cells on PCL/chitosan than that on PCL nanofibers.

The same authors [48] blended Poly(hydroxybutyrate-cohydroxyvalerate) (PHBV) and collagen in two different ratios, and performed electrospinning to fabricate composite PHBV/Col75:25 and PHBV/Col50:50 scaffolds. The major aim of their study was optimizing the solution concentration and electrospinning conditions of these nano-scaled fibrous scaffolds, composed of PHBV, a low cost synthetic material with good mechanical properties, and collagen, the most abundant nerve ECM protein characterized by high costs and the weak mechanical strength. The tensile strength and Young’s modulus of aligned nanofibers along the circumferential direction resulted higher compared to their respective random nanofibers; in addition the aligned nanofibers showed anisotropic behavior, whereby these values in the direction of circumferential stress was significantly higher compared to the values obtained along the axial direction of stress. Moreover, the tensile strength of aligned PHBV/Col50:50 (6.34_0.35MPa) fabricated in this study was even higher than the tensile strength of aligned PCL/gelatin scaffolds fabricated by Ghasemi-Mobarakeh *et al*. [29]. No significant changes in the morphology of PHBV fibers were observed after *in vitro* degradation for 30 days period, and no higher extent of nanofibers breakages were observed for PHBV/Col scaffolds, showing a slow degradation rate compatible with nerve regeneration. After PC12 cell culture, they observed that cell proliferation on composite PHBV/Col nanofibers was better than on pure PHBV nanofibers. Moreover, immunostaining studies highlighted that the cells cultured on aligned PHBV/Col scaffolds showed bipolar extensions with neurite extension parallel to the fiber orientation, while the cells showed multipolar phenotype on the random nanofibers. The authors hypothesized that this behavior could be explained as the preferential alignment of nerve cells on aligned nanofibers, which architecturally mimic the Bungner band formation and guide axonal regeneration.

Biophysical (nanofiber alignment) and biochemical cues (addition of bFGF or EGF) were combined by Lam *et al*. [49]. They compared to different methods of immobilization of bFGF and EGF onto nanofibers: PLLA with adsorbed or heparin immobilized bFGF or EGF. As shown by immunofluorescent staining, the first method was not effective in immobilization of bFGF and EGF onto nanofibers functionalized with heparin promoted axon growth, indicating that the bioactivity of bFGF and EGF was preserved through heparin binding but not by adsorption. The presence of a natural bioactive factor, although modifying the degradation rate and decreasing the mechanical properties of synthetic nanofibers, enhances axon outgrowth and cell proliferation. Several natural-natural polymers have been studied as artificial nerve conduits, in fact natural polymers such as silk fibroin (SF) and collagen have been demonstrated to be suitable as delivery system for bioactive factors in neural tissue engineering.

SF has a slow degeneration rate, shows good mechanical properties, cell biocompatibility and has been demonstrated to support peripheral nerve regeneration [60]. Madduri *et al*. [50] carried out a research on SF conduits loaded with glial cell line-derived neurotrophic factor (GDNF) and nerve growth factor (NGF) and topographically functionalized with aligned and non-aligned SF nanofibers. Chicken embryonic dorsal root ganglion (DRG) and spinal cord (SpC) explants were employed to assess the impact on sensory and motor neurons, respectively, and on the associated glial cells of growth factor release and fiber orientation. They found that fiber alignment played an instructive role to guide neuronal and glial growth parallel to the fibers. In addition, the growth rate was significantly higher when the explants were cultured on SF membranes loaded with GDNF and NGF and structured with the aligned nanofibers. Axonal growth was hardly noticeable with DRG/SpC cultured on unloaded SF scaffold. Thus, that functionalized SF conduits resulted to be suitable for both sensory and motor nerve regeneration and concluded that these constructs hold promise to enhance functional recovery of injured peripheral nerves.

In addition, collagen-glycosaminoglycan (GAG) scaffolds may share similar biological properties with the ECM of the tissue they are replacing, but unfortunately they often show poor mechanical strength. Traditional collagen-GAG in gel or sponge, which have been found to increase biological interactions with cells and speed up tissue regeneration, exhibit scarce mechanical properties and not ideal physical structure. However, an improvement of mechanical characteristics and the alignment of the fibers are expected to lead to a closer simulation of the body environment, mimicking the native ECM [51,61,62].

Timnak and co-workers [51] developed biodegradable random orientated and aligned nanofibrous porous collagen-GAG scaffolds by electrospinning procedure. Oriented collecting of the fibers resulted to improving the tensile strength of the scaffold and, at SEM observation, both SK-N-MC cells and fibroblasts adhered and elongated themselves along the alignment direction of the nanofibers. Cell parallel organization on orientated scaffolds suggested also in this study the superiority of aligned nanofibers, as compared to random scaffolds, in guiding cell morphology and outgrowth. Proliferation rate of both the two aforementioned cell lines in the crosslinked collagen-chondroitin-6-sulfate (C6S) scaffold was determined by MTT assay, in order to determine most cytocompatible crosslinking among four tested. The release rate of C6S was analyzed through ion chromatography assay and C6S was found to enhance biological interactions with cells and motivate tissue regeneration. In accordance with Madduri’s study [50], the combination of the topographical features and the releasing of bioactive factors within nerve conduits composed of natural materials resulted to enhance the quality of these devices in promoting tissue regeneration.

### 2.3. *In Vivo* Studies

Electrospun nanofiber scaffolds have been largely investigated *in vitro*, but only a few studies employed electrospun nanofiber conduits to promote regeneration of peripheral nerves *in vivo*.

Studies performed in animals are usually based on a sciatic nerve injury model, consisting in the positioning of a construct between the proximal and the distal stumps to repair an at least 10 mm nerve gap after rat sciatic nerve transection.

Random electrospun nanofiber scaffolds have been applied for the regeneration of peripheral nerve injuries. Initial studies conducted by Bini’s group in 2004 [52] examined the flexibility and the biological performance of random electrospun poly(l-lactide-co-glycolide) (PLGA) biodegradable nanofiber conduits for peripheral nerve regeneration in rat sciatic nerve model with a 10 mm gap length. They observed no inflammatory response and no tube breakage. Additionally, one month after the surgical approach, five out of eleven rats showed successful nerve regeneration, even of no characterization of functional recovery was provided.

Panseri *et al*. [26] used flexible tubular electrospun scaffolds obtained from a polymer blend of PLGA and poly(ɛ-caprolactone) (PCL) in order to regenerate a 10 mm nerve gap in a rat sciatic nerve model. A base of PCL electrospun microfibers was expected to assure mechanical stability and elasticity and a coating of PLGA/PCL electrospun nanofibers to add more substrate surface for cell attachment, prevent invasion of ectopic cells and allow nutrient exchange. Four months after surgery, in all rats of the control samples (transected sciatic nerve or 10-mm nerve gap left between the transacted stumps) nervous tissue did not reconnect the two stumps of transected sciatic nerves. In most of the treated animals, the inner lumens of the electrospun constructs were filled with regenerated nervous fascicles and neurite outgrowth was found to be mainly oriented along the longitudinal conduit axis. In addition, widespread myelination of the regenerated fibers and collagen IV deposition were detected. Functional neuronal reconnection was also demonstrated by conducting electrical impulses and supporting retrograde transport of fluorescent tracers. Electromyography tests revealed that 70.6% of the fibrous tube-treated rats presented compound muscular action potentials (CMAP), while no CMAP was detected in non-treated animals. However, the found lower nerve conduction rate, bigger F-peak latency and smaller amplitude of the detected cMAP in treated nerves compared to the healthy contralateral nerves, indicating a relatively early phase of nerve regeneration.

Wang and co-workers [53] tested different materials in rat sciatic nerve model (10 mm gap): chitosan nano/microfiber mesh tubes with a deacetylation rate (DAc) of 78% or 93%; bilayered tubes with a nano/microfiber mesh inner structure with a DAc of 78% or 93% and a film outer layer with a DAc of 93%; and film tubes with a DAc of 93%. Isografting was also performed as a control. Although the functional recovery of motor activity was delayed in each group, sensory function recovered first in the control group, followed by the sample treated with tubes with a DAc of 93%. From this study the limits of natural electrospun conduit employment as nerve guide constructs emerged clearly, due to the weak mechanical strength of these materials. However, chitosan nano/microfiber mesh tubes with a DAc of 93% seemed to have sufficient mechanical properties to preserve tube space and to provide a suitable scaffold for neural tissue engineering, with good permeability for cell migration and nutrients diffusion.

The same authors [54] also examined with the same *in vivo* model a funzionalized bilayered chitosan tube, composed of an outer layer of chitosan film and an inner layer of chitosan nonwoven nano/microfiber mesh. They introduced glycine spacers into the CYIGSR sequence, domain of laminin-1, and these bioactive peptides were covalently bound to the chitosan tube. The histological analysis, conduced 5 and 10 weeks after tube implantation, revealed better performances for the funzionalized bilayered chitosan tubes containing the bioactive peptide with the major number of glycines. In fact, bilayered chitosan tube, on which the YIGSR peptide and 6 glycines were added, exhibited efficacy similar to that of the isograft (control).

A few *in vivo* studies have investigated electrospun conduits composed of axially aligned micro/nanofibers.

Wang et co-workers [55] examined a chitosan bilayered nanofiber mesh tubes of randomly oriented electrospun nanofibers in the outer layer and axially aligned electrospun nanofibers in the inner layer. They demonstrated the capability of this device to promote myelination and axonal maturation in a 10 mm rat sciatic nerve transection model. Moreover, functional and electrophysiological recovery was detected.

The use of aligned electrospun fibers in rat sciatic nerve model (10 mm gap) was also investigated by Xie and co-workers [14]. This group carried out a series of preliminary studies that used multi-layered, nanofiber-based conduits, with randomly oriented electrospun nanofibers in the outer surface and axially aligned electrospun nanofibers in the inner surface, similar to those used by Wang *et al*. [55]. Multilayered constructs were found to be capable of supporting nerve regeneration and preserving the quantity, quality, and maturity of axons regenerating across the nerve defect 8 weeks post-operatively. In addition to the histological studies, this group also performed quantitative analysis of conducted CNAP and evoked muscle force amplitude, demonstrating that multi-layered, nanofiber-based NGCs provided superior functional recovery to standard silicone NGCs.

Zhu *et al*. [56] developed a one-step electrospinning process to fabricate a tubular nanofibrous nerve conduit composed of two fully integrated layers, composed of an inner layer with longitudinally aligned nanofibers and an outer layer with randomly organized nanofibers. They demonstrated the long tern (up to 12 months) biological performances of this scaffold in a rat sciatic nerve transection model with random nanofibrous conduits and autografts as controls. 2-month and 12 month histomorphometry analysis revealed that both aligned nanofibrous nerve conduit and autograft groups displayed higher frequencies of large axons and of thick myelin sheaths than the random nanofibrous nerve conduit group. Both aligned nanofibrous nerve conduit and autograft performed significantly better than random nanofibrous nerve conduit, with no statistical difference between aligned electrospun tubes and autografts, as confirmed by 12-month electrophysiological analysis. The results from behavior tests are in line with CMAP amplitude and conduction velocity data.

Kim and colleagues [32] stacked in a 3D configuration both randomly-oriented and aligned electrospun fiber (~400–600 nm in diameter) sheets into polysulfone to fabricate conduit for nerve repair in a 17 mm rat tibial nerve transection model. They found that aligned, but not randomly oriented, polymer fiber constructs successfully promoted axon regeneration, reinnervating muscles, and reforming new neuromuscular junctions, as verified through immunohistochemical and histomorphometric analysis, and behavioral and electrophysiology tests carried out after 16 weeks to measure functional recovery. These findings highlighted the importance of topographic directional cues, deduced by the comparable performances of aligned fibrous constructs and autografts in reducing the functional deficits after nerve injury.

The use of aligned biofunctional electrospun scaffolds was evaluated in an *in vivo* experiment by Chew and colleagues in a 15 mm gap rat sciatic model [57]. The bilayered construct, containing an inner layer of PCLEEP (copolymer of caprolactone and ethyl ethylene phosphate) electrospun microfibers aligned longitudinally or circumferentially to the conduit and an outer layer of PCLLEP film, presented the encapsulation of Glial cell-Derived Neurotrophic Factor (GDNF). Three months after surgery, the samples treated with oriented fiber scaffolds were found to have a higher content of myelinated axons and an improved electrophysiological recovery compared to the group treated with tubes without electrospun fibers. In addition, the bioactive factor further enhanced nerve regeneration and functional recovery. Finally, in a recent study, Hu and colleagues [58] adopted a different *in vivo* model, consisting in a 5-mm facial nerve defect in rats. They performed morphologic and functional evaluations three months after conduit implantation, finding positive outcomes for both electrospun silk fibroin (SF) nanofiber scaffold construct and autograft. They concluded that electrospun SF grafts might be an alternative to autografts in peripheral nerve regeneration.

Altogether, these preliminary studies demonstrate that the use of electrospun micro- and nanofiber scaffolds may represent a promising approach in the treatment of peripheral nerve injury. However, while there are in literature many *in vitro* studies regarding the potentiality of electrospun nanofiber conduits and the superiority of nanostructured devices to support cell attachment and proliferation, there is a lack of information about the use of nanofiber tubes applied to guide and promote neurite outgrowth and functional recovery *in vivo*. Additional investigations in animal models should be carried out to clarify the advantages derived by the use different nanofibers, the orientation of the electrospun fibers (aligned nanofibers are largely demonstrated to better promote neurite outgrowth and direct neurite extension *in vitro*), the mechanical properties, and the control of the pore structure. Moreover, it should be determined if topographical cues were sufficient to enhance nerve regeneration even in the absence of biochemical cues, such as the addition of bioactive molecules.

## 3. Self-Assembling Nanofibers for Neural Tissue Engineering

### 3.1. Method of Production and Major Characteristics

Self-assembling peptide nanofiber scaffold (SAPNS) represents another promising approach to nanofiber fabrication in the field of neural bioengineering [27,63] Biocompatible and bioactive small molecules capable of self-assembling and degradate over time into predictable metabolites are ideal building blocks for scaffolds to regenerate tissues and organs. Of the major biological building blocks—sugars, amino acids and nucleic acids-amino acids offer the widest variety of functionality and cell signaling capacity with rapid and easy synthesis of complex molecules. In other words, self assembling nanofibrous scaffold consists in the spontaneous assembly of ionic self-complementary oligopeptides into patterns or structures without human intervention [64]. Small peptides can be rationally designed to self-assemble into a variety of supramolecular nanostructures, such as hydrogel, spheres, cylinders, tubes, and many other morphologies. Research on implantable materials for tissue regeneration has been primarily focused on biodegradable polymers and more recently synthetic proteins. Currently various types of hydrogels are of great interest in this field.

These scaffolds could indeed form hydrogels when sapeptide solution was exposed to physiological media or salt solution.

Sapeptides generally show hydrophilic heads with hydrophobic tails or, alternatively, periodic repeats of alternating ionic hydrophilic and hydrophobic amino acids, with both molecular types spontaneously forming β-sheet structures. They are isobuoyant in aqueous solution and readily transportable to different environments. Upon exposure to neutral pH aqueous solutions, ions screen the charged peptide residues and hydrophobic residues (constituting the nonpolar surfaces of β-sheets) of different β-sheets can pack together thanks to their hydrophobic interactions in water, thus giving double-layered β-sheet nanofibers, structures that are found in silk fibroin from silkworms and spiders, or β-sheets assemblies parallel to cylindrical nanofibers. Remarkably, sapeptide scaffolds, with great than 99% water content, show nanostructures resembling those of ECM derived substrates. Since the building blocks of self-assembling peptide scaffolds are natural amino acids and their degradation products can be reused by the body, unlike most of the other synthetic biomaterials, they have been shown to elicit a negligible immune response and poor inflammatory reaction in *in vivo* experiments [65]. While simple to produce, chemically crosslinked hydrogels have several shortcomings, such as limited biodegradation, potentially toxic monomers and crosslinking agents, and shrinkage of the hydrogels after crosslinking. The alternative approach that has emerged in the last decade has been to focus on supramolecular nanostructures using fully degradable small molecules.

Non-covalent bonds, such as van der Waals forces, hydrogen bonds, and electrostatic forces mediate in self-assembling process: different proteins and peptides can produce stable and ordered nanofiber structures [27]. Moreover self-assembled nanofibers diameter are smaller at least two magnitudes than electrospun fibers. This process creates nanofibers of the smallest scale (5–8 nm), the fabrication process is a challenging technique, limited to a few polymers, and can only create short fibers with lengths of one to several μm [23,66].

Arginine(R)-Alanine(A)-Aspartate (D) (RAD16-I and RAD16-II) sequences are the most commonly used Sapeptides for neuronal cells culture. (Table 4).

RAD16-I has a modulus of one based on the formula of (RADA)*n*, whereas RAD16-II has a modulus of two based on the formula (RARADADA)*n*.

Some different functional motifs could be added to RAD16, like RGD (Arginine-Glycine-Aspartic acid), based sequences from fibronectin (RGDS Arginine-Glycine-Aspartic Acid-Serine) and from collagen VI (PRGDSGYRGDS), laminin derived motifs IKVAV (Isoleucine-lysine-valine-alanine-valine), BMHP (bone marrow homing peptides) and bioregulatory mediator peptide from the family of myelo-peptides (GFLGFPT). Two other motifs, BMHP1 (SKPPGTSS) and BMHP2 (PFSSTKT), which belong to a family of peptides (bone marrow homing peptides), have been shown enhanced survival of the neural stem cells [67].

Isoleucine-lysine-valine-alanine-valine (IKVAV), which is known to promote and direct neurite outgrowth is another used sapeptide in SAPNSs for neural regeneration studies [68] (Table 5).

### 3.2. *In Vitro* Studies

SAPs have the function to support neurite outgrowth and neural tissue regeneration, as evidenced by Holmes *et al*. [69], RAD16-I and RAD16-II SAPs scaffolds with individual fibers of approximately 10–20 nm in diameter. Experiments were conducted by using several types of neuronal cells, including transformed cell lines (NGF-treated Rat PC12, NGF-preprimed PC12, human SY5Y neuroblastoma) and freshly isolated primary cells (mouse cerebellar granule neurons, mouse hippocampal neurons, rat hippocampal neurons). A number of mammalian cell types could attach to SAPs scaffolds. The NGF treated cells projected extensive neurites that follow the contours of the SAPs scaffolds. In contrast, control cells without NGF treatment did not project neurites on the SAPs scaffolds. Sapeptide scaffolds support extensive neurite outgrowth from cerebellar granule neurons prepared, dissociated mouse and rat hippocampal neurons also attach and project neurites on sapeptide scaffold.

The extracellular matrix (ECM) is a complex mixture of proteins and polysaccharides that provides structural support and domains for cell adhesion, among many other roles. Major components of the ECM include fibronectin, laminin, collagen, chondroitin sulfate, and others. Fibrous materials that display common cell-binding epitopes, including RGDS (Arginine-Glycin-Aspartic Acid-Serine), YIGSR (Tyrosin-Isoleucin-Glycin-Serine-Arginine), and IKVAV (Isoleucine-lysine-valine-alanine-valine), are currently under intense study as ECM mimetics due to their dual roles as structural and adhesive frameworks. Zou *et al*. [70] studied the capacity of supporting neurite outgrowth of SAPs scaffolds by linking FGL motif to RADA-16 (FGL-NS). This novel peptide nanofiber scaffold FGL-NS was compared to RADA-16. The diameter of the fiber of FGL-NS was 38.2 ± 2.7 nm while the diameter of nanofibers of RADA-16 was 16.9 ± 2.3 nm. Rat dorsal root ganglions (DRG) were isolated from adult male Sprague-Dawley rats. The ability of the SAPs to induce neurite outgrowth was evaluated 24 and 48 h post cell seeding. DRG were able to generate neurites on both substrates. The level of neurite growth was significantly higher on FGL-NS than RADA-16. Significantly, longer neurites were observed on FGL-NS, which further confirmed the role of designer self-assembling nanofiber scaffolds containing FGL motif in promoting neurite outgrowth.

Different studies [68,71] support the capacity of sapeptide scaffolds combining functional motifs to promote cell differentiation: Gelain *et al*. [71] showed that Bone Marrow Homing Peptide 1 (BMHP1) functional motif can foster neural stem cell differentiation and stabilize the β-sheet structures found in RADA16-I nanofibers, when linked, via an oligo-glycine spacer, to the RADA16-I self assembling “core”. The study describes a novel ensemble of SAPs, developed from the BMHP1 (BMHP1-SAPs), that spontaneously assemble into tabular fibers, twisted ribbons, tubes and hierarchical self-assembled sheets, organized in the nano and microscale structures. Thirty-two sequences were designed and evaluated, including biotinylated and unbiotinylated sequences, as well as a hybrid peptide-peptoid sequence. To assess the potential of the BMHP1-SAPs for cell cultures and regenerative medicine, Gelain *et al*. cultured human neural stem cells (hNSCs) *in vitro* over scaffolds. BMHP1-SAPs fostered hNSC survival, spreading, and differentiation.

Silva *et al*. [68] used murine neural progenitor cells (NPCs) to study *in vitro* the use of a self-assembling artificial scaffold to direct cell differentiation. The molecular design of the scaffold incorporated the pentapeptide epitope IKVAV, which is found in laminin and is known to promote neurite sprouting and to direct neurite growth. The nanofibers had high aspect ratio and high surface areas, 5 to 8 nm in diameter and with lengths of hundreds of nanometers to a few micrometers. The cells survived the self-assembling process and remained viable during the time of observation. There was no significant difference in viability between cells cultured on poly(d-lysine) (PDL, a standard substrate used to culture many cell types) relative to cells encapsulated in the nanofiber network to SAPs scaffold. These results suggest that diffusion of nutrients, bioactive factors, and oxygen through these highly hydrated networks is sufficient for survival of large numbers of cells for extended periods of time. In the bioactive scaffolds, cell body areas and neurite lengths of NPCs that had differentiated into neurons showed statistically significant differences with respect to cells cultured on PDL- or laminin-coated substrates. Neurons within the nanofiber networks were noticeably larger than neurons in control cultures. The average cell body area of encapsulated progenitor cells in the networks was significantly greater after 1 and 7 days. Encapsulation in the nanofiber scaffold led to the formation of large neurites after only 1 day, whereas cells cultured on PDL and laminin had not developed neurites at this early time. The neurons also had significantly longer processes in the scaffolds compared with cells cultured on the PDL substrates after 7 days.

Over and above cell differentiation, the cell proliferation was improved by Zou *et al*. with a novel sapeptide material combining a functional motif. Zou *et al*. [72] using the RADA-16 peptide nanofiber as a base material, specifically selected a new functional motif to design a novel self-assembling scaffold. The FRM motif, a synthetic fibroblast growth factor receptor (FGFR) ligand derived from the first fibronectin type III domain of neural cell adhesion molecule. The FRM motif was attached to RADA16 for the development of a novel peptide scaffold (FRM-NS). RADA-16 could form stable β-sheet structure and undergo self assembly to form nanofibers, 16.5 ± 2.6 nm in fiber diameter. Similar nanofibers also formed with the functionalized designer peptide scaffold. The diameter of the fiber self-assembled by peptide RADA-FRM was 36.3 ± 4.4 nm Although FRM-NS had no effect on differentiation of neural stem cells (NSCs), it could improve NSCs proliferation in comparison to the pure RADA-16 peptide scaffold. NSCs were seeded on the top-surface of each SAPs scaffold. Compared to RADA-16, FRM-NS showed a significantly higher cell proliferation. Furthermore, FRM-NS scaffold stimulated NSCs migration into the SAPNSs scaffold, suggesting NSCs encapsulated in FRM-NS could migrate to the injured zone *in vivo*. However, FRM-NS scaffold had no obvious effect on the differentiation of NSCs into neurons, which should be further improved.

3-D sapeptide scaffolds combining functional motifs could be promoting vehicle for neural cell culture as demonstrated by Gelain *et al*. [67]. The research reported the use of a designer peptide nanofiber scaffolds to produce 3-D cultures for the study of mouse adult neural stem cells. They synthesized 18 different peptides that directly incorporate various functional motifs with the self-assembling peptide RADA16. These motif sequences have shown to promote cell adhesion, differentiation and bone marrow homing activities. These functionalized peptides self-assemble into nanofiber scaffolds where cells could be fully embedded by the scaffold in 3-D. The self-assembling peptide RADA16 was appended with different motifs: RGD based sequences from fibronectin (RGDS), from collagen VI (PRGDSGYRGDS), laminin derived motifs (YIGSR, IKVAV, PDSGR), bone marrow homing peptides (BMHP1 and BMHP2) and myelo-regulatory peptide (GFLGFPT). The seeded mouse neural stem cells exhibit higher levels of attachment on some of the tested scaffolds, with the best viability and survival found on Matrigel, natural extract considered as the most effective and standard cell-free substrate for neural stem cell culture and differentiation, RADA16-BMHP1 and RADA16- BMHP2. The cells in RADA16-BMHP1 and RADA16- BMHP2 designer peptide scaffolds exhibited neuronal branching similar to that found in the Matrigel. RADA16-BMHP1 and RADA16-BMHP2 designer peptide scaffolds influenced the mouse neural stem cell differentiation nearly on par with heterogeneous Matrigel.

3D scaffold has been investigated by Cunha *et al*. [73]. They used RADA16-I-based SAPs that incorporated the ubiquitin receptor binding site RGD (Arg-Gly-Asp) functional motif (RADA16-RGD) and also the laminin-derived motifs BMHP1 (RADA16-BMHP1) and BMHP2 (RADA16-BMHP2), together with pure RADA16-I. The aim was to seed and culture NSCs in a 3D biomaterial scaffold and to determine the best conditions for their proliferation and differentiation. For all the scaffolds analyzed, NSC proliferation seemed to be dependent and inversely related to the scaffold concentration, so that higher proliferation rates were obtained with lower SAP concentrations. The best scaffold for NSC proliferation seemed to be RADA16-BMHP1 and RADA16-BMHP2, although a statistically significant difference was not obtained. After proliferating within the scaffolds for 5 days, NSCs were able to differentiate into the three major neural cellular phenotypes: neurons, astrocytes, and oligodendrocytes, and the higher number of differentiated cells was obtained for RADA16-RGD, which demonstrated that this scaffold performs the best with regard to maintaining NSC staminality. These results indicate that functional motifs are able to direct NSC *versus* proliferation or differentiation.

Koutsopoulos *et al*. [74] investigated different SPNSs scaffolds: (i) ac-(RADA)4-CONH2; (ii) ac-(RADA)4-GG - BMHP1 - CONH_2_; (iii) ac-(RADA)4-GG - BMHP2 - CONH_2_; (iv) ac-(RADA)4-GG - RGDS - CONH_2_. For the 3-D neural tissue cultures, freshly passaged neural stem cells (with added human FGF-2 to increase neural stem cell differentiation to neural progenitors but without EGF) were mixed with SAPNSs scaffolds, Matrigel or Collagen I solutions. Encapsulation of neural stem cells and subsequent tissue cultures in (RADA)4-BMHP2, (RADA)4-BMHP1 and (RADA)4-RGDS peptide hydrogels showed that cell viability was similar or less compared to that of the non functionalized ac-(RADA)4-CONH2 peptide. 3-D tissue cultures in SAPNs scaffolds represent a more realistic system compared to traditional 2-D studies and 3-D models that employ animal derived materials such as Matrigel and Collagen I. Results show that during the first 2 weeks of culture, neural stem cells in Matrigel appear to proliferate, differentiate and form processes better than in SAPNSs. However, this effect of Matrigel is limited to the initial growth period and the situation is reversed over time. When neural tissue cultures were investigated for longer periods of time, better cell survival rates in SAPNs scaffolds compared to Matrigel and Collagen I was observed.

It is shown that neural stem cells can be encapsulated successfully in hydrogel matrices of SAPNs scaffolds and present marked differentiation into projection neurons, astrocytes and oligodendrocytes. Those observations prompted inquiry into the functionality and mechanism of interaction between the SAPs functionalization motifs and the neural cell types in the tissue cultures. Main features of the above *in vitro* studies are summarized in Table 6.

### 3.3. *In Vivo* Studies

Animal models have been used to investigate the application of SAPNSs for treatment of Peripheral nerve system (PNS) injuries.

Rat sciatic nerve transection is the peripheral nerve injury model used to test self-assembled nano fibrous scaffold.

Zhan *et al*. [63] examined a novel artificial nanofiber nerve conduit for peripheral nerve regeneration. Conduit consists in 12mm long aorta filled with SAPNS solution made of RADA16-I peptide. The right sciatic nerve was exposed in 36 adult Sprague–Dawley (SD) female rats and a 10 mm gap was made at middle part of the nerve trunk. Animals were randomly divided into three groups: (1) artificial nanofiber nerve conduit (*n* = 18); (2) empty nerve conduit (*n* = 13); (3) defect without treatment (*n* = 5). Conduits were implanted to repair the nerve gap after sciatic nerve transection. The study suggested that nanofiber conduit connected the nerve gap with excellent peripheral nerve-like appearance without any atrophy. The artificial nerve implant integrated with host nerve, which was evidenced by the smooth transition of both connection zones without any significant scarring occurrence. In contrast, modest axonal regrowth appeared in control implant (blood vessel without any filling). The NCAP (nerve compound action potentials) obtained by electrophysiology proved that the nerve conduction got through the artificial nerve implant. The mean amplitude of *n*CAP was significant greater in test group than in control group.

## 4. Perspectives in Oral and Maxillofacial Surgery

Nerves injuries in oral and maxillofacial area can be treated in several ways: external decompression, internal neurolysis, excision of neuroma, neurorrhaphy, nerve graft, nerve sharing, “guided” nerve regeneration, neurectomy, nerve capping, and nerve redirection. These microsurgical techniques are not required in case of neuropraxia due to the spontaneous functional recovery. In case of axonotmesis, surgical treatment could be necessary when lack of sensation or dysesthesias is reported. When trigeminal nerve branches are transacted (neurotmesis), the surgical approach is recommended for the restoration of sensory function and the reduction or elimination of pain [75].

In oral and maxillofacial surgery, nerves mainly involved in injuries are the branches of the trigeminal and the facial nerves. Facial, inferior alveolar and lingual nerves are probably the most frequently damaged. Lingual and facial nerves transaction could be repaired by direct neurorrhaphy immediately after the mobilization of the nerve stumps in the soft tissues. Tension free inferior alveolar nerve neurorrhaphy is difficult to perform unless the incisive nerve transaction, which could expose the patient to the risk of formation of a stump neuroma and causes loss of sensory in lower incisive teeth and mandibular labial gingiva [15].

When the clinician is not able to re-connect the two stumps, the inter-positioning of a nerve graft (autograft, allograft, xenograft, alloplastic graft) is required to bridge the gap. The most commonly used autografts are the sural nerve and the greater auricular nerve. Autografting, however, presents many disadvantages, such as multiple surgeries, loss of function at the harvested site, limited availability of donor nerves, diameter discrepancy and fascicular mismatch between the donor and recipient nerves. Also autologous vein could be employed as nerve graft [76].

Alternative conduits for nerve repair are cadaveric nerve allografts, which are related to a risk of immunological reaction and disease transmission [17].

The application of guided nerve regeneration in form of microsurgically applied conduits of various material have been proposed for trigeminal nerve injuries, as artificial nerve guidance conduits. Many synthetic conduits are available to treat nerve defect injuries and are generally divided in two main groups: non-resorbable materials (e.g. silicone, gore-tex) and biodegradable synthetic materials (e.g., polyglycolic acid, poly(dl-lactide-e-caprolactone), semipermeable collagen type I). Resorbable materials seem to obtain superior results than non-resorbable ones, avoiding the risk of foreign body reactions due to scar formation, compression and demyelination associated with permanent tubing [77]. To the best of our knowledge, in the current literature there is a substantial lack of studies regarding the application of nanoconduits for nerve regeneration *in vivo,* in oral and maxillofacial surgery. As previously reported, Hu *et al*. [58] used electrospun silk fibroin (SF) nanofiber scaffolds to repair a 5-mm facial nerve defect in rats, obtaining results comparable to autografts in peripheral nerve regeneration.

It is possible to hypothesize that the use of novel eletrospun nanostructured conduits, applied with the same surgical protocols performed till now, may enhance repair outcomes.

In addition, considering the anatomical characteristics of mandibular canal, the inferior alveolar nerve is difficult to visualize and repair without performing a large mandibular osteotomy. In light of such considerations, there is a need for a more conservative approach. We hypothesize a novel application of self-assembled nanofiber scaffold, to be injected into the mandibular canal, which can retain the material.

## 5. Conclusions

A promising strategy to promote axon regeneration is guided nerve regeneration employing novel nano-structured biomaterials. In this view, several *in vitro* studies have investigated electrospun nanofiber scaffold for nerve regeneration. At this time, to the best of our knowledge, just a few *in vivo* studies have applied electrospun nanomaterial guided nerve regeneration in rat sciatic models (10 to 17 mm gap). Results are promising, consisting of better performances for resorbable aligned electrospun biosynthetic materials, but nowadays clinical reports and studies are still lacking.

The majority of studies regarding Sapeptide nanofibrous scaffolds for nerve regeneration highlights the limitations of this technique to produce scaffolds with adequate mechanical properties. For this reason, self-assembled scaffolds have been tested in animal models for central nervous system regeneration. For peripheral nervous system regeneration, A novel SAPNS-enhanced scaffold artificial nerve with artery segment serving as conduit in a rat sciatic nerve model was proposed to overtake the mechanical limitation.

Electrospun conduit utilization for facial, lingual and inferior alveolar nerves tubulization might be considered for repair of injuries, within the limitations given by the difficult surgical approach in the oral and maxillofacial area.

## Figures and Tables

**Table 1.** Acronyms and definition of the most common used synthetic materials used in nanostructured electrospun scaffolds.

Acronym	Definition
PAN-MA	poly acrylonitrile-*co*-methylacrylate
PCL	poly(ɛ-caprolactone)
PCLEEP	copolymer of caprolactone and ethyl ethylene phosphate
PDS	polydioxanone
PES	polyethersulfone
PGA	poly(glycolic acid)
PHB	poly(3-hydroxybutyrate)
PHBV	poly(3-hydroxybutyrate-*co*-3-hydroxyvalerate)
PHT	poly(3-hexylthiophene)
PLCL	poly(l-lactide-*co*-caprolactone)
PLGA	poly(lactide-*co*-glycolide)
PLLA	poly(l-lactic acid)
PPG	poly(propylene glycol)

**Table 2.** Main features of the electrospun materials used in *in vitro* studies.

References	Electrospun materials	Fiber orientation	Diameter of the fibers (nm)	Culture
Yang *et al*., 2004 [38]	PLLA	Random	272 ± 77	neural stem cell line C17.2
Yang *et al*., 2005 [39]	PLLA	Random Aligned	Average diameter: RANDOM 250 (1 wt%) 1500 (3 wt%) ALIGNED 300 (2 wt%) 1500 (5 wt%)	neural stem cell line C17.2
Corey *et al*., 2007 [40]	PLLA	Random Intermediate Aligned	524 ± 305 (range: 150–1540)	rat dorsal root ganglia (DRG); human neuroblastoma cell lines (SH-EP and SH-SY5Y)
Chow *et al*., 2007 [31]	PDS	Random Aligned	Not reported	rat dorsal root ganglia (DRG); rat astrocytes
Subramanian *et al*., 2012 [41]	PLGA/PHT	Random Aligned	RANDOM 196 ± 98 ALIGNED 200 ± 80	rat Schwann cells (CRL-2765)
Qu *et al*., 2013 [37]	Silk fibroin (SF)	Random	400 ± 76 800 ± 37 1200 ± 117	rat subventricular zone (SVZ)-derived neurons; rat astrocytes
Wang *et al*., 2012 [42]	Tussah silk fibroin (TSF)	Random Aligned	400 ± 67 800 ± 35	human embryonic stem cells (hESCs)-derived neural precursors (NPs)
Wang *et al*., 2011 [43]	Collagen	Random Aligned	ALIGNED 694 ± 157 RANDOM 785 ± 177	rat spinal cord derived neural progenitor cells (NPCs)
Koh *et al*. 2008 [28]	PLLA/laminin	Random	100–500	PC 12 cells
Christopherson *et al*., 2009 [44]	PES/laminin		238 ± 45 749 ± 153 1452 ± 312	rat hippocampus-derived adult NSCs (rNSCs)
Ahmed *et al*., 2006 [45]	Polyamide/neuroactive peptides derived from human tenascin-C	Random	180	(rat) cerebellar granule neurons, cerebral cortical neurons, hippocampal and ventral spinal cord neuronal cultures, dorsal root ganglia (DRG)
Xie *et al*., 2009 [46]	PCL/laminin	Random Aligned	Not reported	primary dorsal root ganglia (DRG)
Ghasemi-Mobarakeh *et al*., 2008 [29]	PCL/gelatine	Random Aligned	PCL: 431 ± 118 PCL/gelatin 50:50: 113 ± 33, PCL/gelatin 70:30: 189 ± 56	neural stem cell line C17.2
Prabhakaran *et al*. 2008 [47]	PCL/chitosan	Random	PCL: 630 ± 40 Chitosan: 450 ± 48 PCL/chitosan: 190 ± 26	rat Schwann cell (RT4-D6P2T)
Prabhakaran *et al*., 2013 [48]	PHBV PHBV/collagen (50:50; 75:25)	Random Aligned	**RANDOM** PHBV: 472 ± 85 PHBV/Col(75:25): 266 ± 60 PHBV/Col(50:50): 260 ± 60 **ALIGNED** PHBV: 386 ± 74 PHBV/Col(75:25): 205 ± 50 PHBV/Col(50:50): 229 ± 65	PC 12 cells
Lam *et al*., 2010 [49]	PLLA PLLA + basic Fibroblast Growth Factor (bFGF) or Epidermal Growth Factor (EGF) PLLA + heparin + bFGF or EGF	Random Aligned	Not reported	human embryonic stem cell (ESC)–derived neural cells
Madduri *et al*., 2010 [50]	silk fibroin silk fibroin + glial cell line-derived neurotrophic factor (GDNF) + nerve growth factor (NGF)	Random Aligned	400–500 nm	chicken embryonic dorsal root ganglions (DRG) and spinal cord (SpC)
Timnak *et al*., 2011 [51]	collagen + chondroitin-6-sulfate (C6S)	Random Aligned	50–350 nm	SK-N-MC human neuroblastoma cell lines and human fibroblast

**Table 3.** Main features of the electrospun materials used in *in vivo* studies. Legend: IL: inner layer; OL: outer layer.

References	Electrospun materials	Fiber orientation	Diameter of the fibers (nm)	Nerve injury model	Gap (mm)
Bini *et al*., 2004 [52]	PLGA	Random	-	Rat sciatic nerve	10
Panseri *et al*., 2008 [26]	PCL PLGA/PCL	Random	PCL: ~2500–8000 PCL/PLGA: 140–500 (279 ± 87)	Rat sciatic nerve	10
Wang *et al*., 2008 [53]	Chitosan	Random	DAc of 93%: peak <200 and has a downward-sloping distribution of diameters DAc of 78%: there is a single peak in the distribution centered around 400–600	Rat sciatic nerve	10
Wang *et al*., 2008 [54]	Chitosan + YIGSR laminin-1 sequence linked to 2 different glycine Spacers	Random	700 ± 502	Rat sciatic nerve	10
Wang *et al*., 2009 [55]	Chitosan	Aligned (IL) + Random (OL)	most distributed under 400	Rat sciatic nerve	10
Zhu *et al*., 2011 [56]	PLCL/PPG	Aligned (IL) + Random (OL)	Not reported	Rat sciatic nerve	10
Kim *et al*., 2008 [32]	PAN-MA	Aligned	400–600	Rat tibial nerve	17
Chew *et al*., 2007 [57]	PCLEEP + Glial cell-Derived Neurotrophic Factor (GDNF)	Aligned	5080 ± 50	Rat sciatic nerve	15
Hu *et al*., 2013 [58]	Silk fibroin (SF)	-	-	Rat facial nerve	5

**Table 4.** Two different RAD16 sequences.

Sequence	Formula
RAD16-I	+ − + − + − + − AcN-RADARADARADARADA-CNH_2_
RAD16-II	+ + − − + + − − AcN-RARADADARARADADA-CNH_2_

**Table 5.** List of motifs that could be added to Arginine(R)-Alanine(A)-Aspartate (D) (RAD16-I and RAD16-II) sequences.

Motif	Composition
RGD	Arginine-Glycine-Aspartic Acid
RGDS	Arginine-Glycine-Aspartic Acid-Serine
PRGDSGYRGDS	from collagen VI
IKVAV	Isoleucine-lysine-valine-alanine-valine
BMHP	bone marrow homing peptides
SKPPGTSS	BMHP1
PFSSTKT	BMHP2
YIGSR	Tyrosin-Isoleucin-Glycin-Serine-Arginine
GFLGFPT	bioregulatory mediator peptide from the family of myelo-peptides
FGL	synthetic fibroblast growth factor receptor (FGFR) ligand derived from neural cell adhesion molecule (NCAM)
FGL-NS	synthetic fibroblast growth factor receptor (FGFR) ligand derived from neural cell adhesion molecule (NCAM) + RADA16
FRM	synthetic fibroblast growth factor receptor (FGFR) ligand derived from the first fibronectin type III domain of neural cell adhesion
FRM-NS	synthetic fibroblast growth factor receptor (FGFR) ligand derived from the first fibronectin type III domain of neural cell adhesion + RADA16

**Table 6.** *In vitro* studies on self assembling materials: main features.

References	Self-assembled material	Cells type	Diameter of the fibers(nm)
Holmes *et al*., 2000 [69]	RAD16-I RAD16-II	PC12 cells; mouse cerebellar granule neurons; mouse hippocampal neurons; rat hippocampal neurons	10
Gelain *et al*., 2006 [67]	RAD16-I + motifs	Adult mouse neural stem cells	10
Zou *et al*., 2010 [70]	FGL-NS RADA16	Rat dorsal root ganglions (DRG)	38.2 ± 2.7 (FGL-NS) 16.9 ± 2.3 (RADA16)
Gelain *et al*., 2011 [71]	RAD16-I + BMHP1 RAD16-I + different BMHP1 derived peptide sequence variations	cultured human neural stem cells	depends on scaffold type
Silva *et al*., 2004 [68]	IKVAV	Murine neural progenitors cells (NPCs)	5–8
Cunha *et al*., 2011 [73]	RADA16 RADA16 + BMHP1 RADA16 + BMHP2 RADA16 + RGD	mice neural stem cells	10 (RADA16) 13–15 (RADA + motifs)
Zou *et al*., 2013 [72]	RADA16 FRM-NS	rat neural stem cells	16.5 ± 2.6 (RADA16) 36.3 ± 4.4 (FRM-NS)
Koutsopoulos *et al*., 2013 [74]	(RADA)4 (RADA)4 + BMHP1 (RADA)4 + BMHP2 (RADA)4 + RGDS	rat neural stem cells	6–10
